# Enhancing cardiomyocyte reprogramming efficiency by targeting cellular senescence is mediated via Rb1 gene

**DOI:** 10.1186/s13287-025-04776-7

**Published:** 2025-12-29

**Authors:** Juntao Fang, Qiangbing Yang, Renée G. C. Maas, Pieter Vader, Michal Mokry, Noortje A. M. van den Dungen, Li Qian, Junjie Xiao, Raymond Schiffelers, Zhiyong Lei, Joost P. G. Sluijter

**Affiliations:** 1 Department of Cardiology, Guangdong Cardiovascular Institute, Guangdong Provincial People’s Hospital (Guangdong Academy of Medical Sciences), Southern Medical University, Guangzhou, 510080 China; 2 Department of Cardiology, Experimental Cardiology Laboratory, University Medical Center Utrecht, Utrecht, The Netherlands; 3 Circulatory Health Laboratory, UMC Utrecht, Regenerative Medicine Center Utrecht, University Utrecht, Utrecht, 3508 GA The Netherlands; 4 CDL Research, University Medical Center Utrecht, Utrecht, The Netherlands; 5 Central Diagnostic Laboratory, University Medical Center Utrecht, Utrecht University, Heidelberglaan 100, Utrecht, 3508 GA The Netherlands; 6 McAllister Heart Institute, University of North Carolina, Chapel Hill, NC USA; 7 Cardiac Regeneration and Ageing Lab, Institute of Cardiovascular Sciences, School of Life Science, Shanghai University, Shanghai, 200444 China

**Keywords:** Direct cardiac reprogramming, Senescence, RB1

## Abstract

**Introduction:**

Direct reprogramming of fibroblasts into cardiomyocytes by overexpressing cardiac transcription factors Gata4, Mef2c, and Tbx5 (GMT) is a promising way for cardiac repair, however, the low reprogramming efficiency remains a significant challenge. Cellular senescence, an irreversible cell-cycle arrest occurring in mitotic cells, has been reported to influence the efficiency of induced pluripotent stem cell (iPSC) reprogramming.

**Methods:**

We established an inducible GMT expression system in mouse embryonic fibroblasts (MEFs) and human fetal cardiac fibroblasts (hFCFs) using the PiggyBac transposon system. RNA sequencing was performed to identify genes associated with cellular senescence during reprogramming. Selected senescence-related genes were knocked down using shRNA, and their impact on reprogramming efficiency was assessed via flow cytometry, gene expression analysis, and staining for senescence and apoptosis markers.

**Results:**

Direct cardiac reprogramming induced cellular senescence and apoptosis, evidenced by enhanced β-Gal staining, elevated expression of senescence markers P16 and GLB1, and increased apoptosis rates. RNA sequencing and gene set enrichment analysis (GSEA) revealed significant upregulation of senescence-related genes (RB1, RBBP4, RBBP7, CBX8, and CDKN1B). Knockdown of these genes, particularly RB1, significantly enhanced reprogramming efficiency, increasing the proportion of GFP + cells in MEFs and α-actinin + cells in hFCFs. RB1 inhibition also reduced senescence marker levels and upregulated endogenous cardiac transcription factors GATA4 and MEF2C.

**Conclusions:**

Our findings demonstrate that cellular senescence might serves as a barrier to direct cardiac reprogramming and offer novel insights into the regulatory mechanisms involved in this process.

**Supplementary Information:**

The online version contains supplementary material available at 10.1186/s13287-025-04776-7.

## Background

Myocardial infarction (MI) is one of the major causes of death worldwide. After MI, the lost myocardium is replaced by fibrotic tissue due to the limited regenerative capacity of cardiomyocytes, which eventually leads to heart failure. So far, there are only a few effective treatments for end-stage heart failure patients which are caused by the massive cell loss post-MI, such as cardiac transplantation and mechanical support devices. Their applications are however limited due to donor availability and various drawbacks including bleeding, infection, thrombosis, and embolism [1]. For this reason, many studies focused on repairing the damaged heart post-MI and re-introduce contractile tissue via cell-based approaches in the infarcted region to maintain and restore myocardial function. One alternative strategy is to restore functional cardiomyocytes through direct cellular reprogramming of terminal differentiated cardiac fibroblast into cardiomyocytes by the overexpression of cardiac transcription factors Gata4, Mef2c, and Tbx5 (GMT) [2]. Several studies have demonstrated that cardiac reprogramming is capable of improve heart function post-MI [2–5], which makes cardiac reprogramming a promising approach in regenerative medicine. The efficiency of cardiac reprogramming, however, remains a major challenge [6] and many approaches have been investigated to identify different genes or conditions that could enhance the cardiomyocyte reprogramming [4, 7–11]. Although some improvements have been made, more in-depth mechanical insights and strategies to further enhance efficiency is warranted.

Cellular Senescence is an irreversible arrest during the G1 transition of the cell cycle, induced by replicative exhaustion or in response to stresses such as DNA damage, chemotherapeutic drugs, and abnormal expression of oncogenes [12]. Cellular reprogramming processes initially triggers a stress response with characteristics of senescence, called reprogramming-induced senescence (RIS), and acts as an initial barrier limiting the efficiency of induced pluripotent stem cell (iPSC) reprogramming [13–15]. The role of cellular senescence in cardiac reprogramming, however, has not been fully investigated yet. In this study, we demonstrate that cardiac reprogramming triggers a massive cellular senescence response, and that the cardiac reprogramming efficiency can be improved by inhibiting cellular senescence via suppressing RB1 in both mouse and human cells.

## Materials and methods

### Generation of inducible cardiac reprogramming reporter in mouse embryonic fibroblasts

Mouse embryonic fibroblasts (MEF) were obtained from the α-MHC/GFP transgenic mouse on embryonic day 13.5, as previously described [2, 5]. In short, hearts were digested with 0.1% trypsin and plated on plastic dishes. Attached fibroblasts were cultured for 7 days and α-MHC-GFP^+^ cells were sorted and cultured in DMEM/M199 medium containing 10% FBS at a density of 10^4^/cm^2^. To generate a MEF GMT cell line, MEFs were transfected with a PiggyBac vector [16] carrying an inducible TetOn3G promotor, the mouse GMT reprogramming factors, and mCherry. The PiggyBac transposon system, which integrates randomly into the genome, as described in Yusa et al. [17]. PiggyBac system’s random integration ensures that the GMT cassette is expressed independently of the endogenous GMT genes, which remain intact. After co-transfection by using Lipofectamine 3000 (Invitrogen, Catalog number: L3000001), the cells were selected with puromycin at a final concentration of 2 µg/ml for 3 days (Gibco, A11138-03) to obtain a stable cell line.

### Generation of inducible cardiac reprogramming human fetal cardiac fibroblast (hFCFs) cell line

Human fetal cardiac fibroblasts (hFCFs) cell line carrying an inducible cardiac reprogramming cassette was created with the PiggyBac (PB) transposon system as described above. hFCFs were transfected with either a GMT vector or an empty vector (EV) together with a transposase plasmid. As a control, the EV-hFCFs cell line was created containing the same TetOn 3G promotor and mCherry tag, but not containing the reprogramming cassette. After co-transfection by using Lipofectamine 3000 (Invitrogen, Catalog number: L3000001), the cells were selected with puromycin at a final concentration of 2 µg/ml (Gibco, A11138-03) to obtain a stable cell line. Due to the tetOn3G promotor, it is possible to activate and inactivate the reprogramming cassette on every preferred time point by adding or removing doxycycline (Dox) [18].

### Cardiac reprogramming induction

Cells were seeded at a density of 5 × 10^4^ cells per well in a 6-well plate coated with 0.1% gelatin and cultured in the reprogramming medium (DMEM with 10% FBS and 1% P/S). Reprogramming medium was changed every other day. Dox (1 µg/ml, Clontech, 631311) was added one day after the cells were plated to start reprogramming and maintained during cardiac reprogramming. The day of Dox administration was considered as day 0.

### Plasmids construction

All shRNAs constructed in the pLKO.1 HIV-based lentiviral vector were purchased from Sigma-Aldrich. The lentivirus packaging and envelop vectors, pCMVR8.74 (#22036) and pCMV-VSV-G (#8454) were obtained from Addgene. The coding sequences of RB1, RBBP4, RBBP7, CBX8 and CDKN1B were PCR amplified from MEF cDNA and cloned into pMXs-puro vector with compatible restriction enzyme sites. Each gene was designed with two pairs of coding sequences. pLKO.1 vector itself was used as the control shRNA. The primers required in this study are listed in supplementary table S1 and purchased from Integrated DNA Technologies.

### Lentivirus packaging, transduction and transfection

Lentivirus packaging was conducted in 293 T cell line. This cell lines were maintained in DMEM growth media containing DMEM with 10% FBS, 1% PS (Penicillin/Streptomycin). One day before transfection, 293 T cells were plated at a density of 6 × 10^6^ cells per T175 flask. The next day, 10 µg shRNA viral vector, 5 µg pCMVR8.74 and 5 µg pCMV-VSV-G were co-transfected to 293 T cells. Supernatant was collected 72 h post-transfection and filtered through 0.45 μm syringe filter (CORNING, 516–1954) and incubated with PEG6000 solution (8% final concentration) overnight at 4°C. The next day, mixtures were centrifuged at 6000 rpm for 30 min at 4 °C, and the pellets were resuspended in 100 µl 50mM Tris-HCl per T175 flask virus. Lentiviruses were freshly added to target cells with 4 µg/ml polybrene (sigma, TR-1003-G) or frozen at −80 °C freezer for future usage. For transfection, MEFs/Hfcfs were plated into gelatin-coated 24 well plates at a density of 2 × 10^4^ cells per well in DMEM medium. The next day, 5–10 µl of lentiviruses expressing shRNAs were added in iCM media (10% FBS of DMEM/M199 (4:1)) for 16 h and changed every 2–3 days followed by selection with 2 µg/ml puromycin for 48 h to obtain stable cell line. At the indicated time points, reprogramming cells were harvested for analyses.

### RT-PCR

Total RNA was isolated at the indicated time points using the Nucleospin RNA isolation kit from Macherey-Nagel (740.955.250). The qScript cDNA synthesis kit from Quanta BioSciences (95047-100) was used to synthesize cDNA from the isolated RNA samples (300 ng per sample). The quantitative real-time polymerase chain reaction (qRT-PCR) was performed with iQ SYBR Green Supermix (Bio-Rad). β-actin was selected as the housekeeping gene and was used for the calculation of normalized gene expression levels (ΔCt). The primers required in this study are listed in supplementary table S2 and purchased from Integrated DNA Technologies.

### NGS RNA sequencing

RNA concentration and integrity were analyzed with the Agilent 2100 Bio analyzer prior to proceeding with sequencing (Agilent RNA 6000 Nano Kit). RNA concentration for each single sample must be at least more than 15ng/ul and the total RNA amount is no less than 0.3ug. The library construction kit is MGIEasy RNA Directional Library Prep Set (1000006385), made in MGI. Sequence on the DNBSEQ-G400 platform with PE150. The sequencing data was filtered with SOAPnuke by (1) Removing reads containing sequencing adapter; (2) Removing reads whose low-quality base ratio (base quality less than or equal to 15) is more than 20%; (3) Removing reads whose unknown base (‘N’ base) ratio is more than 5%, afterwards clean reads were obtained and stored in FASTQ format. The clean reads were mapped to the reference genome using HISAT2. After that, Ericscript (v0.5.5) and rMATS (V3.2.5) were used to detect fusion genes and differential splicing genes (DSGs), respectively. Bowtie2 was applied to align the clean reads to the gene set, a database built by BGI (Beijing Genomic Institute in ShenZhen), in which known and novel, coding and noncoding transcripts were included. Expression level of gene was calculated by RSEM (v1.3.1). Essentially, differential expression analysis was performed using the DESeq2(v1.4.5). These RNA sequencing and data mapping is conducted in BGI Hongkong Tech Solution NGS Lab. Gene expression profiles were analyzed using Omics Explorer 3.2 (Qlucore) and principal component analysis (PCA) was used to visualize the data. Gene set enrichment analysis (GSEA) was also performed with Qlucore to determine if a gene set of interest was significantly enriched in one condition compared to another. Gene sets with (False Discovery Rate [FDR] < 0.1) were considered significantly enriched in the comparison made.

### Fluorescence-activated cell sorting (FACS)

For MEFs, cells from different groups were first washed with PBS, detached by trypsin and collected to 15 ml Eppendorf tube, filtered with Flowmi™ Cell Strainers, 40 μm (southern labware, BAH136800040) and were analyzed by Cytoflex flow cytometer (Beckman Coulter, #A00-1-1102). For hFCFs, cells were washed and fixated in 70% cold ethanol for 30 min. Later, cells were washed twice with PBS and blocked with 5% goat serum for 30 min, washed twice with PBS and stained with primary antibody α-Actinin (Sigma, A7811, 22ug/ml) overnight at 4℃, washed with PBS twice and stained with goat anti-mouse IgG Alexa Fluor 488 s antibody (Invitrogen, A11001, in 1:400 dilution) for one hour at room temperature, and then washed with PBS twice, filtered with Flowmi™ Cell Strainers, 40 μm and analyzed by Cytoflex flow cytometer in the similar setting.

### Cellular apoptosis assay

Cell apoptosis was determined by Annexin V/PI staining. The cells were stained with Annexin V/PI for 15 min in the dark and then analyzed using a flow cytometer (Accuri C6, USA). Annexin V^+^/PI^+^ cells were regarded as apoptotic cells in the late stage. A minimum of 10,000 cells within the gated region was measured by flow cytometry.

### β-Gal staining

To visually compare the senescence among different groups, we performed β-galactosidase staining (β-Gal) according to the manufacturer’s protocol of senescence β-galactosidase staining kit (#9860, Cell Signaling Technology). Briefly, cells were fixed in 1x Fixative Solution for 15 min and washed with PBS twice. Subsequently, cells were treated with galactosidase staining solution and placed in a 37 ℃ incubator overnight. The next day, the blue color (indicative of senescence) occurs, and pictures were taken under microscope.

### Statistical analysis

Data were analyzed with GraphPad Prism 9 and comparisons were performed with t-test (non-parametric tests) and a two-way ANOVA. Data were presented as mean ± SEM. *p* < 0.05 is considered as significantly different.

## Results

### Direct cardiac reprogramming induces cellular senescence and apoptosis

**Fig. 1:**
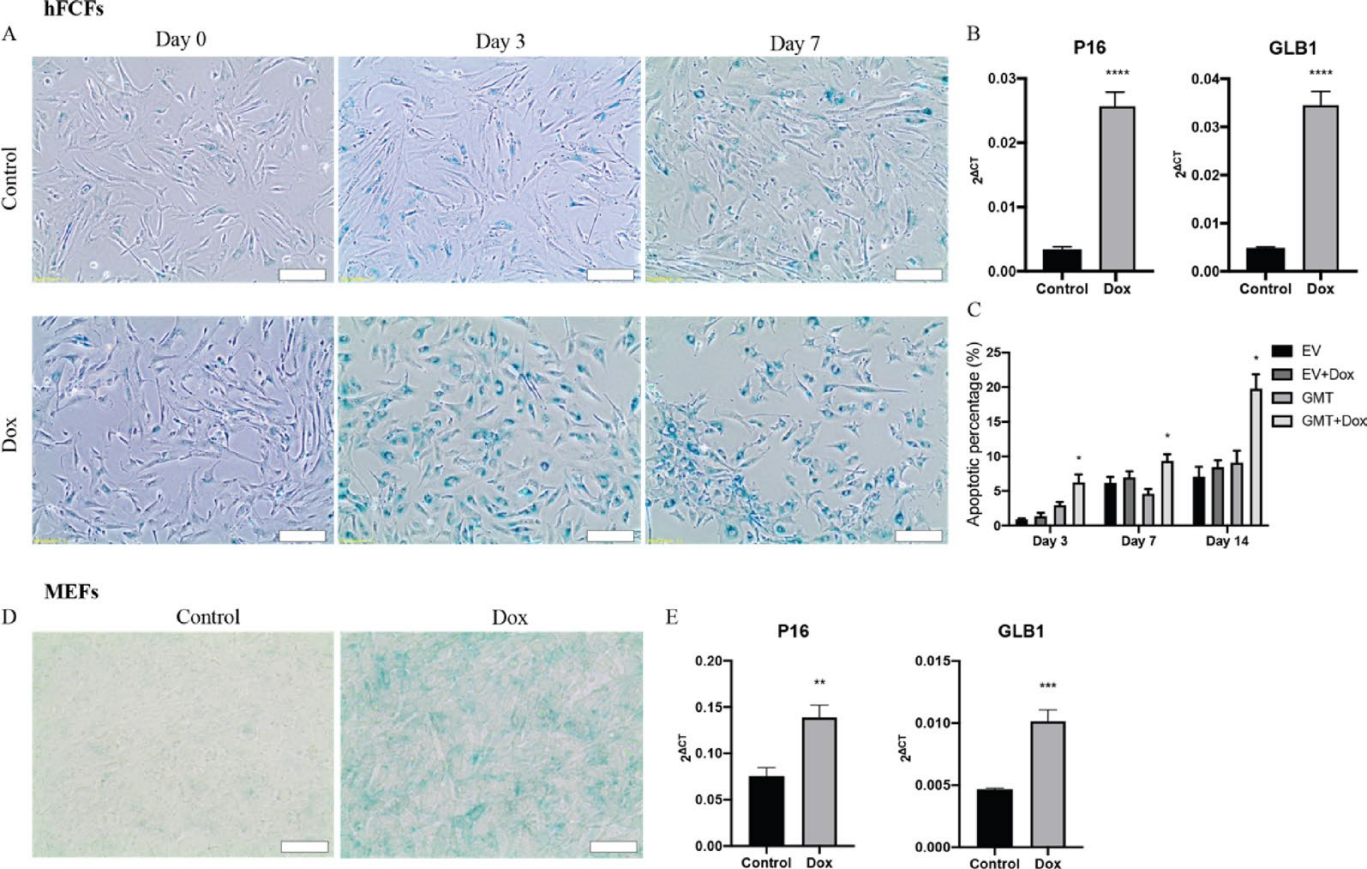
Direct cardiac reprogramming accelerated cellular senescence and apoptosis. **A** Representative images of β-gal staining at different time points during cardiac reprogramming in hFCFs. Scale bar = 200 μm. **B** Relative mRNA expression of senescence markers P16 and GLB1 seven days after initiation of cardiac reprogramming in hFCFs. **C** Cellular apoptosis percentage was evaluated with Annexin V/PI Staining and analyzed by FACS at different time points upon initiation of cardiac reprogramming in hFCFs. **D** Representative images of β-gal staining three days after cardiac reprogramming in MEFs. Scale bar = 200 μm. **E** Relative mRNA expression of senescence marker P16 and GLB1 seven days after reprogramming in MEFs. Mean values + SEM of three independent experiments is shown (*n* = 3). Data were analyzed with two-way ANOVA. *p ≤ 0.05, **p ≤ 0.01, ***p ≤ 0.001 and ****p ≤ 0.0001

The Dox inducible cardiac reprogramming model in both MEFs and hFCFs were established and verified as described previously [19]. After initiation of cardiac reprogramming by Dox exposure, we noticed cells display signs of cell cycle arrest and apoptosis. To investigate whether direct cardiac reprogramming by GMT overexpression induced cellular senescence, we performed β-Gal staining and observed indeed an increased senescence signal, both in MEFs and hFCFs upon initiation of cardiac reprogramming (Fig. 1A, D). To further validate, we also determined the level of senescence markers GLB1 and P16 and observed that their expression level was also significantly increased (Fig. 1B, E). To determine if direct cardiac reprogramming triggers cell apoptosis, Annexin V/propidium iodide (PI) double-staining was used, and the apoptosis rate was analyzed by flow cytometry analyses. We observed that the number of Annexin V^+^/PI^+^ cells increased significantly upon exposure to Dox at different time point (Fig. 1C), indicating that initiation of cardiac reprogramming causes a significant cell death over time.

**Fig. 2:**
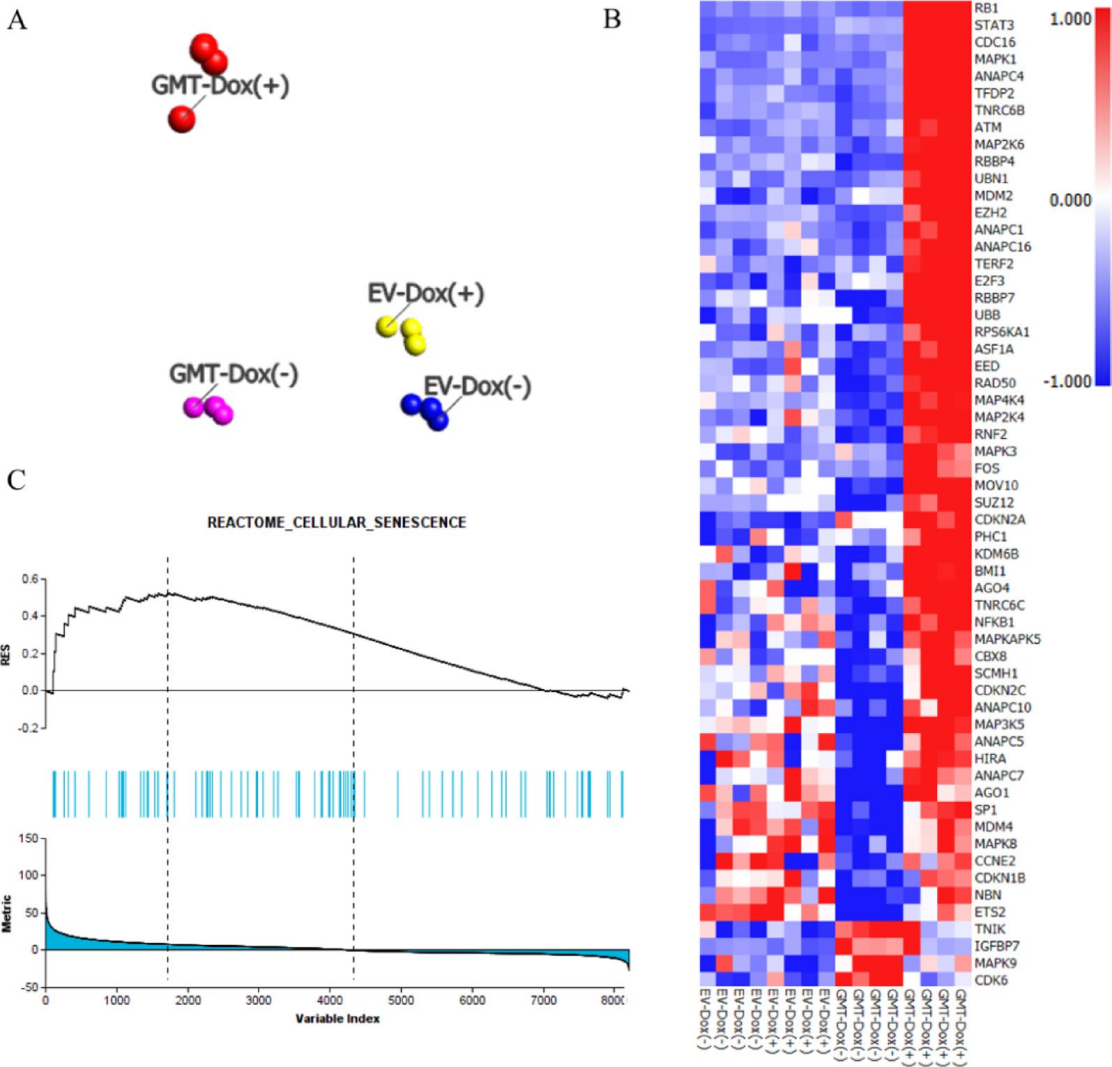
Analysis of differential expression on the RNA-Seq data of hFCFs. **A** PCA plot analysis was based on differentially expressed genes between GMT Dox (+) group and other control groups (FDR = 0.1). **B** Heatmap analyses demonstrated that genes in cellular senescence pathway are significantly upregulated in GMT Dox (+) group compared to control groups (FDR = 0.1). **C** GSEA was performed to elucidate whether the applied gene set is statistically enriched in certain pathways, such as cellular senescence pathway. Data were analyzed through Qlucore Omics Explorer

To further investigate which pathways and genes are involved in direct cardiac reprogramming, we performed RNA sequencing to identify the differentially expressed genes between GMT Dox (+) group and other control groups. Transcriptional differences within these groups were visualized in a PCA plot (Fig. 2A) and, as expected, GMT Dox (+) is distinct from both the GMT Dox (-) as empty vector controls. To investigate the transcriptional differences between these groups further, differential gene expression analysis was performed. Heatmap analyses showed that gene sets related to cellular senescence were significantly upregulated in GMT Dox (+) group in comparison to control groups. This includes genes such as RB1, RBBP4, RBBP7, CBX8 and CDKN1B (Fig. 2B). We performed GSEA to identify statistically enriched gene sets in transcriptomic data between these groups and found that in GMT Dox (+) group a significant enrichment of genes associated with cellular senescence during cardiac reprogramming was present compared to other control groups (Fig. 2C).

### ShRNA screen identified RB1 as a critical modulator of cardiac reprogramming in both hFCFs and MEFs

**Fig. 3:**
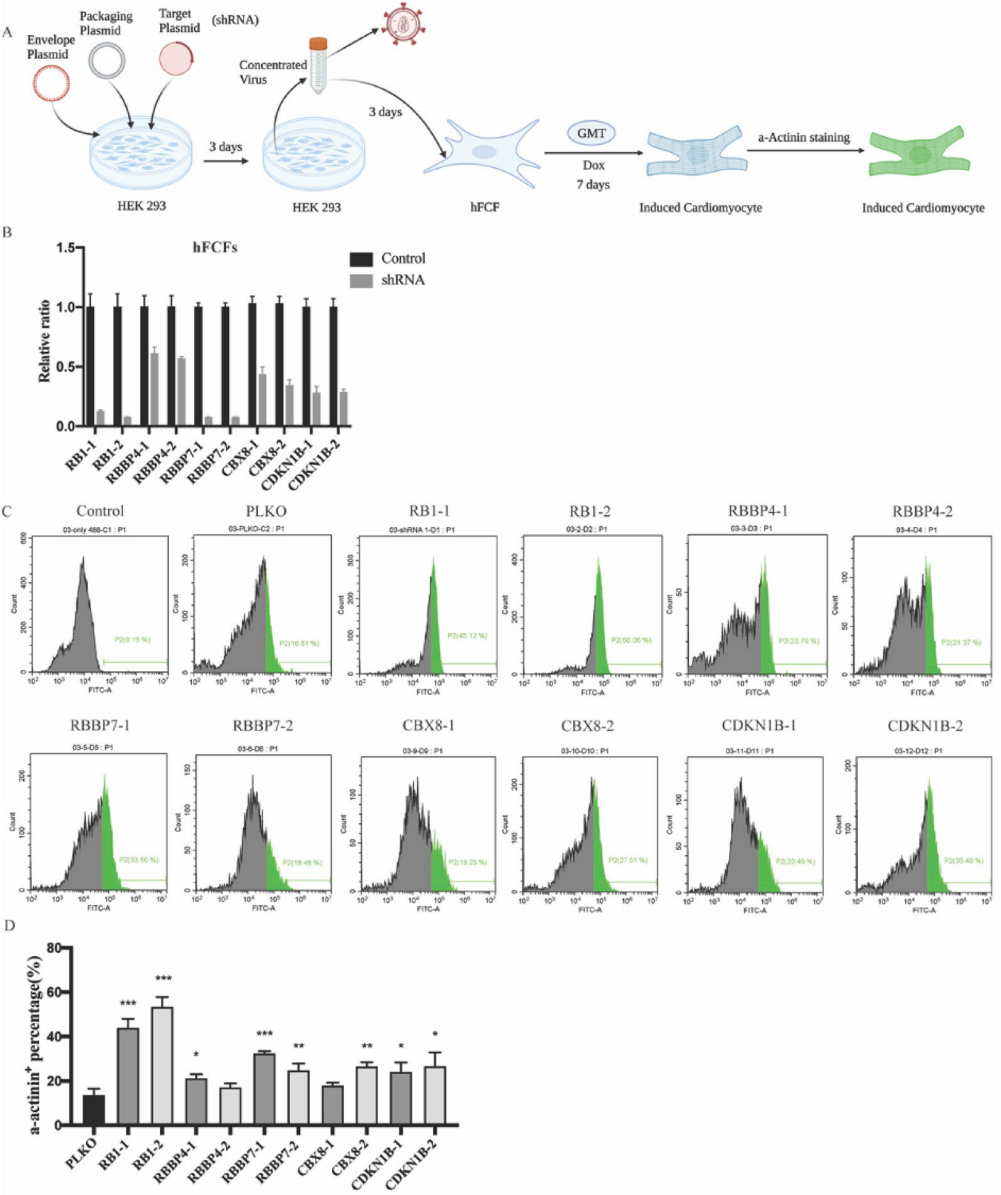
shRNA screen identified RB1 as a critical modulator of cardiac reprogramming in hFCFs. **A** Schematic overview of the shRNA screen for identifying critical regulators of cardiac reprogramming in hFCFs. **B** Knockdown efficiency of indicated shRNAs measured by RT-PCR. Expression values for each gene were normalized to empty vector PLKO infected group. PLKO indicated as shRNA control. **C** Representative FACS image of α-actinin^+^ cells in hFCFs ten days after transduction of lentiviruses encoding shRNAs or PLKO (empty vector). **D** Quantification of α-actinin^+^ hFCFs upon Dox exposure. Mean values + SEM of three independent experiments is shown (*n* = 3). Data were analyzed with two-way ANOVA. *P < 0.05 vs. control; **P < 0.01 vs. control; ***P < 0.001 vs. control; ****P < 0.0001 vs. control

**Fig. 4:**
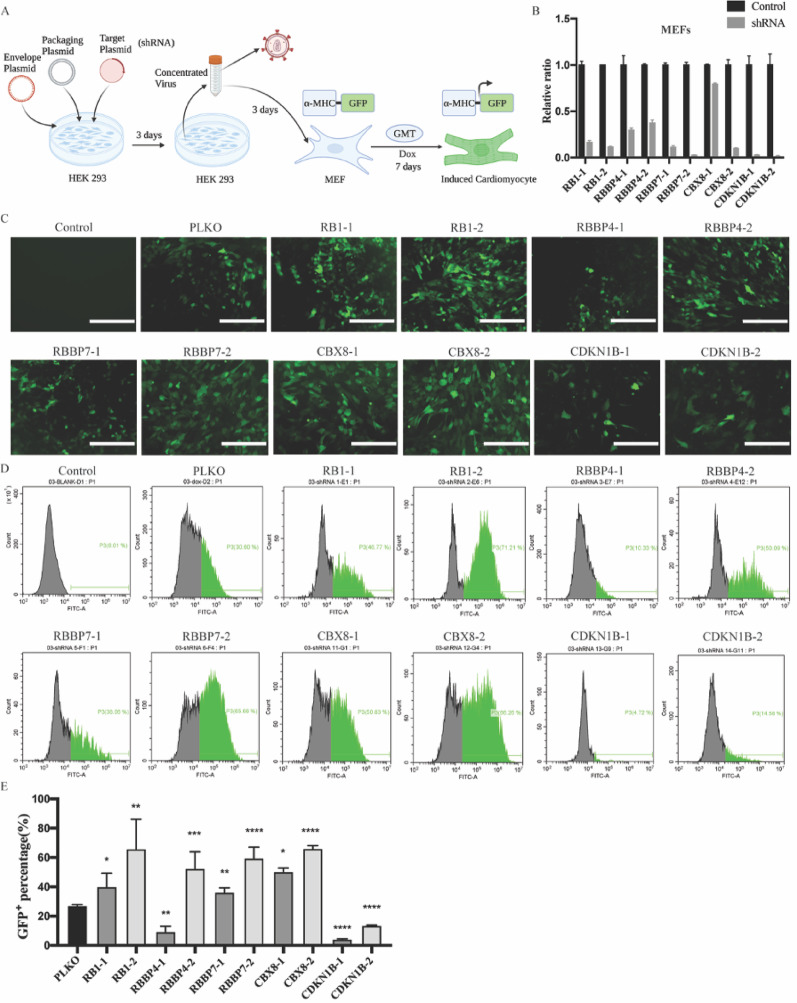
shRNA screen identified RB1 as a critical modulator of cardiac reprogramming in MEFs. **A** Schematic overview of the shRNA screen for identifying critical regulators of cardiac reprogramming in MEFs. **B** Knockdown efficiency of indicated shRNAs measured by RT-PCR. Expression values for each gene were normalized to empty vector PLKO infected group. PLKO indicated as shRNA control. **C** Representative fluorescence images of MEFs with Dox exposure, scale bar 200 μm. **D** Representative FACS image of GFP^+^ cells in MEFs ten days after transduction of lentiviruses encoding shRNAs or PLKO. **E** Quantification of GFP^+^ MEFs upon Dox exposure. Mean values + SEM of three independent experiments is shown (*n* = 3). Data were analyzed with two-way ANOVA. *P < 0.05 vs. control; **P < 0.01 vs. control; ***P < 0.001 vs. control; ****P < 0.0001 vs. control

Given that cellular senescence hampers iPSCs reprogramming and is induced upon cardiac reprogramming, we hypothesize that inhibition of senescence may improve cardiac reprogramming efficiency. To investigate this hypothesis, senescence causal genes RB1, RBBP4, RBBP7, CBX8 and CDKN1B were suppressed in both hFCFs and MEFs with shRNA constructs as indicated in Figs. 3A and 4A, respectively. For each candidate, a pool of two independent shRNA constructs that target different regions within the gene were used and the knockdown efficiency was validated by RT-PCR (Figs. 3B and 4B), the expression of each target gene was normalized to the negative control. Individual shRNA pools were then transduced into MEFs, and the GFP signal was clearly visible when treated for 7 days with Dox (Fig. 4C). These initial results were further validated by flow cytometry by quantifying the percentage of GFP^+^ cells. Although to various degrees, RB1, RBBP4, RBBP7 and CBX8 inhibition led to a significant increase in the proportion of GFP^+^ in MEFs (Fig. 4D, E). To determine if the finding that senescence impairs successful cardiac reprogramming is applicable in hFCFs, we specifically knocked down these genes in hFCFs with shRNA and observed that RB1, RBBP7, CBX8 and CDKN1B inhibition displayed a consistent increase in α-actinin^+^ cells proportion detected by FACS (Fig. 3C, D). Based on these results, both RB1 and RBBP7 inhibition seem to contribute to a significant increase in reprogramming efficiency of both MEFs and hFCFs. Since RB1 inhibition showed a consistent and more robust cardiac-inducing effect and has been also reported to facilitate iPSCs reprogramming [20], we selected RB1 as our target gene for subsequent follow-up study.

### Inhibition of RB1 ameliorates cellular senescence, promote cardiac reprogramming efficiency via de-suppressing the expression of endogenous cardiac transcription factors GATA4 and MEF2C

**Fig. 5:**
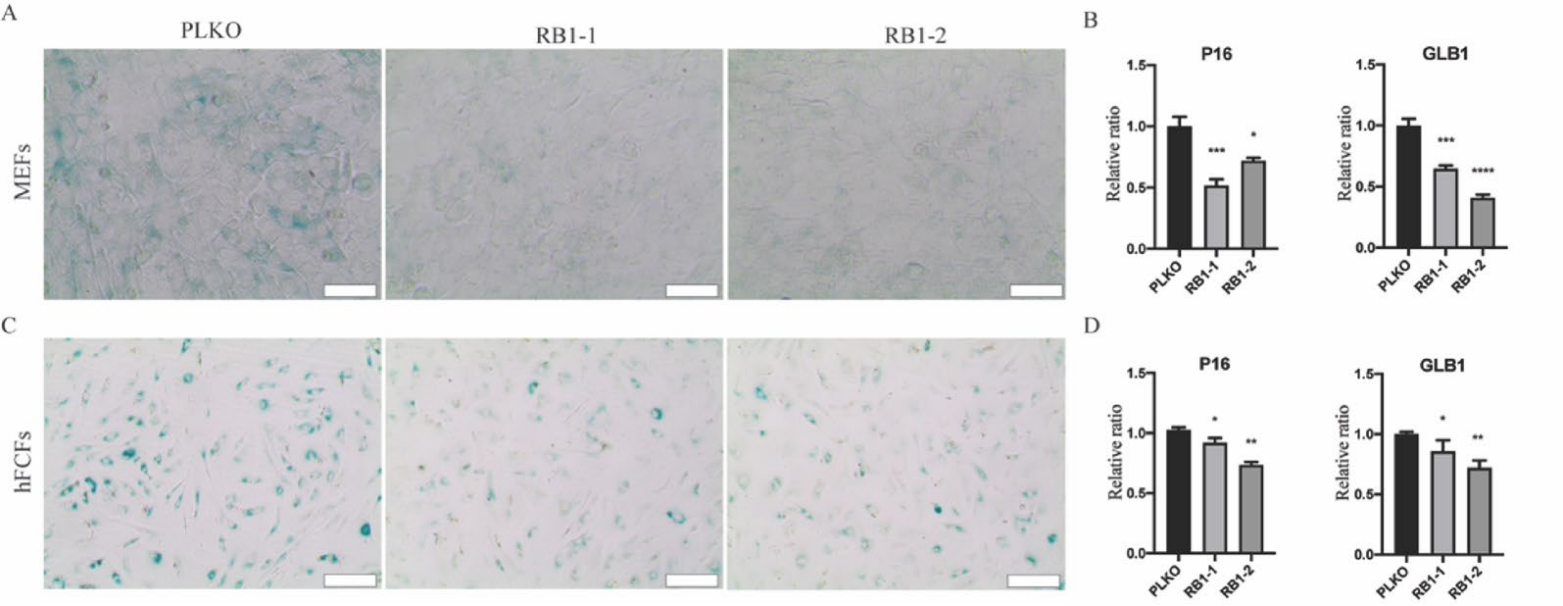
Knock down of RB1 ameliorates cellular senescence. **A** Representative images of β-gal staining three days after cardiac reprogramming with RB1 inhibition in MEFs. **B** Relative mRNA expression of senescence marker P16 and GLB1 seven days after cardiac reprogramming with RB1 suppression in MEFs. **C** Representative images of β-gal staining three days after cardiac reprogramming with RB1 inhibition in hFCFs. **D** Relative mRNA expression of senescence marker P16 and GLB1 seven days after cardiac reprogramming with RB1 suppression in hFCFs. Mean values + SEM of three independent experiments is shown (*n* = 3). Data were analyzed with two-way ANOVA. *P < 0.05 vs. control; **P < 0.01 vs. control; ***P < 0.001 vs. control; ****P < 0.0001 vs. control

**Fig. 6:**
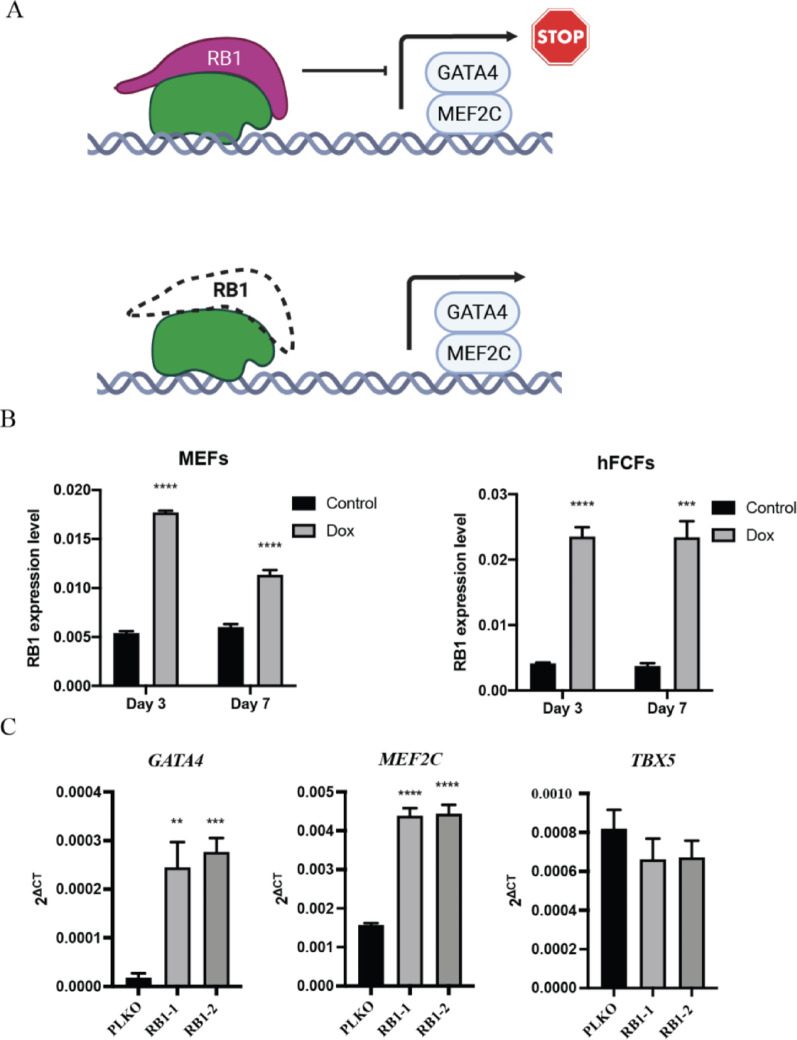
RB1 represses the expression of GATA4 and MEF2C in hFCFs. **A** Schematic overview how RB1 potentially influences the expression of GATA4 and MEF2C. **B** Relative mRNA expression of RB1 in MEFs and hFCFs during cardiac reprogramming at different time point. **C** Relative mRNA expression of *Gata4*, *Mef2c*, and *Tbx5* after RB1 suppression in hFCFs. Mean values + SEM of three independent experiments is shown (*n* = 3). Data were analyzed with two-way ANOVA. *P < 0.05 vs. control; **P < 0.01 vs. control; ***P < 0.001 vs. control; ****P < 0.0001 vs. control

To further confirm that RB1 is responsible for the cellular senescence, we measured the expression level of senescence marker P16 and GLB1 with RT-PCR and performed β-Gal staining as well after RB1 silencing. We observed that RB1 inhibition led to a significant reduction in senescence, as indicated by β-Gal staining in MEFs (Fig. 5A) and hFCFs (Fig. 5C) during cardiac reprogramming and associated with a significant decrease in P16 and GLB1 expression in MEFs (Fig. 5B) and hFCFs (Fig. 5D). In order to explore how the absence of the RB1 protein might render MEFs more amenable to reprogramming, as RB1 is a transcriptional suppressor (Fig. 6A), we found that RB1 expression increased significantly during cardiac reprogramming (Fig. 6B) and also determined the consequence of RB1 loss on endogenous TF gene expression. The PiggyBac system drives exogenous GMT expression while endogenous genes are separately regulated. Interestingly, we observed a significant increase in endogenous GATA4 and MEF2C expression in RB1-deficient MEFs as determined by RT-PCR (Fig. 6C), whereas TBX5 expression is not affected.

## Discussion

In this study, we observed that direct cardiac reprogramming by increasing GMT factors induces a massive cellular senescence response in both mice and human fibroblasts. To explore the role of senescence in cardiac reprogramming, we performed RNA-seq and observed that a significant set of genes were enriched that are linked to cellular senescence pathways, confirming our morphological observations. Subsequently, several genes were selected from this pathway and suppression of these genes via shRNA constructs obtained higher direct cardiac reprogramming efficiency both in mouse fibroblasts and human fibroblasts. Specifically, among these identified genes, we found that RB1 acted as a major barrier during cardiac reprogramming, and we demonstrated that RB1 removal could further increase efficient iCM generation. A significant increase in OCT4 and SOX2 expression in MEF and human fibroblasts was previously been shown in response to RB1 knockdown during iPSCs reprogramming [20]. In order to explore how RB1 inhibition facilitate MEFs more amenable to direct cardiac reprogramming, we determined the consequence of RB1 loss on gene expression and found a significant increase in GATA4 and MEF2C expression in RB1-deficient MEFs. This suggest that a loss of RB1 could enhance cardiac reprogramming via further forced expression of GATA4 and MEF2C, which is consistent with previous studies that GATA4 and MEF2C are critical to heart development [21–23], whereas Tbx5 was not affected. In addition to RB1, we also identified a set of regulators including RBBP4, RBBP7 and CBX8 that function as facilitators to cardiac reprogramming and were also reported involved in epigenetic modification which is crucial to cardiac reprogramming [24–26], this will stimulate follow-up studies to further explore the role of these factors in cardiac reprogramming.

The capacity of RB1 to prevent cardiac reprogramming raises the question of whether RB1 can be used directly for cardiac regeneration. Previous research showed that RB1 plays a critical role in tumor suppression by promoting cell cycle exit, maturation, and maintenance of the terminally differentiated state in cardiomyocytes [27, 28]. In the context of cell cycle regulation and postmitotic maintenance, RB1 expression is minimal or absent in embryonic hearts (e.g., at E11.5 in mice), facilitating the proliferative expansion of cardiac progenitors [29]. RB1 expression increases in neonatal CMs and becomes prominent in adults, driving cell cycle withdrawal and terminal differentiation by suppressing E2F-dependent genes associated with G2/M progression and cytokinesis [29]. Additionally, RB1 and p130 interact with heterochromatin protein 1-γ (HP1-γ) at H3K9-trimethylated promoters, ensuring robust gene silencing and preventing cell cycle reentry [30]. In adult CMs, the combined loss of RB1 and p130 leads to derepression of these genes, promoting proliferation without altering global histone modifications [30]. Alam et al. demonstrated that inhibition of RB1, along with actively expressed cell senescence inducers such as Meis2, effectively induced terminally differentiated, postmitotic, adult cardiomyocytes to re-enter the cell cycle. These re-activated adult cardiomyocytes acquired anti-apoptotic and proangiogenic properties, leading to improved cardiac function following myocardial infarction. Notably, induced adult cardiomyocyte proliferation was only possible with simultaneous inhibition of RB1 and overexpression of Meis2; separate manipulation of these genes did not result in much proliferation [31]. This finding is consistent with the study by Hatzistergos et al., which showed that RB1 and CDKN2A cooperatively regulate cell-cycle progression and differentiation during cardiomyocyte development and repair [32]. Overall, these findings suggest that RB1 inhibition may be a potential therapeutic strategy for cardiac regeneration.

Our study is the first to systematically conduct a loss-of-function screen targeting senescence-associated genes to improve cardiac reprogramming efficiency. Building on Sun et al. [33], which highlighted fibroblast aging and senescence markers, we demonstrate that targeted suppression of senescence effectors, particularly RB1, can overcome this barrier to enhance reprogramming, and the feasibility of an RNAi-mediated functional screen to identify key barriers of cardiac reprogramming. Furthermore, the platform described in this study could be used to develop high-throughput large-scale loss-of-function screens. In combination with other approaches such as single-cell omics, it will be possible in the near future to identify each barrier during cardiac reprogramming and remove the roadblocks to enhance cardiac reprogramming.

Certain limitations should be acknowledged, Doxycycline (DOX), used to initiate the reprogramming system, could potentially influence our results. Future studies employing alternative systems, such as viral delivery, could further validate these findings. Secondly, the proposed transcription repression-based mechanism, inferred from increased GATA4 and MEF2C expression following RB1 knockdown, lacks direct evidence of RB1 binding to these genes. Although the literature supports RB1’s role as a repressor of cell cycle and differentiation-related genes [34], which aligns with our observation of increased GATA4 and MEF2C expression upon RB1 silencing. Future studies, such as chromatin immunoprecipitation (ChIP) experiments, are needed to confirm direct RB1 binding and validate this mechanism.

In summary, our study revealed that silencing senescence effectors could be a sufficient strategy to improve cardiac reprogramming efficiency. It is crucial to understand how senescence affects cardiac reprogramming and investigate novel approaches to overcome the negative impact of senescence on cardiac reprogramming. In addition, defining the precise timing in which temporary inhibition of senescence is required to enhance cardiac reprogramming will be the next step. Despite our encouraging observation regarding senescence inhibition-mediated enhanced cardiac reprogramming, we merely focus on in vitro models in our study, further studies are required to investigate the effect in vivo. We believe that senescence will be a key process to target to explore more efficient strategies for promoting tissue repair and regeneration.

## Supplementary Information

Supplementary Material 1.

## Data Availability

The datasets generated during and/or analyzed during the current study are not publicly available but are available from the corresponding author on reasonable request.

## Abbreviations

CBX8: Chromobox 8
CDKN1B: Cyclin dependent kinase inhibitor 1B
Dox: Doxycycline
DSGs: Differential splicing genes
EV: Empty vector
FACS: Fluorescence-activated cell sorting
GMT: Gata4 Mef2c Tbx5
GSEA: Gene set enrichment analysis
hFCFs: Human fetal cardiac fibroblasts
iCMs: Induced cardiomyocytes
iPSC: Induced pluripotent stem cell
MEFs: Mouse embryonic fibroblasts
MI: Myocardial infarction
PCA: Principal component analysis
RB1: Retinoblastoma gene
RBBP4: RB binding protein 4
RBBP7: RB binding protein 7
RT-PCR: Real-time polymerase chain reaction
